# Photodynamic inactivation strategies for maximizing antifungal effect against *Sporothrix* spp. and *Candida albicans* in an *in vitro* investigation

**DOI:** 10.1371/journal.pntd.0012637

**Published:** 2024-11-12

**Authors:** Amanda Regina Rocha, Natalia Mayumi Inada, Ana Paula da Silva, Vanderlei Salvador Bagnato, Hilde Harb Buzzá

**Affiliations:** 1 PPG Biotec, Federal University of São Carlos, São Carlos, Brazil; 2 São Carlos Institute of Physics, University of São Paulo, São Carlos, Brazil; 3 Department of Biomedical Engineering, Texas A&M University, College Station, United States of America; 4 Institute of Physics, Pontificia Universidad Catolica de Chile, Santiago, Chile; Albert Einstein College of Medicine, UNITED STATES OF AMERICA

## Abstract

**Background:**

Sporotrichosis is a zoonotic disease caused by the dimorphic fungus *Sporothrix* spp., leading to skin lesions that can, in some cases, progress and result in the death of infected individuals. *Candida albicans* is another fungus involved in several skin, oral, and vaginal mucosal infections. Fungal diseases are concerning due to increasing incidence and the limited variety of antifungal classes available for treatment. Furthermore, antifungal medications can cause various side effects, exacerbated by their prolonged use during infection treatment. There is a need to explore alternatives to conventional drugs that are effective, fast, and safe in combating sporotrichosis. This study aimed to achieve *in vitro* elimination of the fungi *Sporothrix brasiliensis* and *Sporothrix schenckii* through Photodynamic Inactivation (PDI), using curcumin as a photosensitizer and in combination with antifungal agents used in the treatment of sporotrichosis.

**Methodology:**

Yeasts of *Candida albicans*, *Sporothrix brasiliensis*, and *Sporothrix schenckii* were subjected to Photodynamic Inactivation (PDI) using light at a wavelength of 450 ± 10 nm, irradiance of 35 mW/cm^2^, delivering a fluence of 31.5 J/cm^2^, with curcumin as the photosensitizer at doses ranging from 0.75 to 150 μg/mL. After determining the Minimum Inhibitory Concentration (MIC) values of the antifungal drugs itraconazole, ketoconazole, and potassium iodide, sub-MIC doses of these antifungals were combined with sub-MIC doses of curcumin in a new PDI session.

**Conclusion:**

Photodynamic inactivation is a promising technique in the treatment of sporotrichosis, as well as its combination with antifungals. The combination of curcumin in concentrations ranging from 0.75 g/mL a 7.5 g/mL with sub-MIC concentrations of itraconazole, ketoconazole, and potassium iodide was able to completely inactivate the fungi *C*. *albicans*, *S*. *brasiliensis* and *S*. *schenckii*, indicating that PDI may increase the effectiveness of antifungals. However, further studies are needed to establish protocols for future clinical applications.

## Author summary

Fungal infections, notably sporotrichosis caused by *Sporothrix* spp., pose increasing challenges due to their infectious nature and limited treatment options. These infections are particularly concerning in immunocompromised individuals, resembling *Candida albicans* in their severity and potential for fatal outcomes. Treatment typically relies on oral antifungals, which are often difficult to administer effectively.

Photodynamic Inactivation (PDI) emerges as a promising approach to enhance antifungal therapy. PDI combines a photosensitizing agent with specific wavelengths of light, generating reactive oxygen species that effectively target and eliminate microbial cells. In this study, we investigated PDI in conjunction with subinhibitory concentrations of curcumin and antifungal drugs (itraconazole, ketoconazole, potassium iodide) against *Candida albicans*, *Sporothrix brasiliensis*, and *Sporothrix schenckii*.

Our results demonstrated that even at curcumin levels below inhibitory thresholds and antifungal concentrations as low as half the Minimum Inhibitory Concentration (MIC), PDI successfully eradicated these pathogens. This synergistic approach not only highlights PDI’s efficacy as a precursor to antifungal treatment but also suggests potential benefits in reducing drug concentrations and associated side effects.

Overall, this study underscores PDI’s role as a potent adjunct therapy in combatting fungal infections, offering a promising strategy to improve treatment outcomes and manage infections caused by these challenging fungi.

## Introduction

Fungal infections have raised concerns as they are responsible for increased mortality in hospitalized patients [1]. The high cost and toxicity of existing antifungal drugs make it challenging to use these medications [2], and the emergence of resistant strains that lead to infectious diseases will be considered the main causes of death in the coming years [3].

The fungus that most commonly causes disease in humans is *Candida* spp., with the capacity to infect both healthy and hospitalized individuals, including those immunocompromised. *Candida albicans* is involved in several diseases, such as skin, oral, and vaginal mucosal infections [4]. Furthermore, infections caused by *Candida* species have been associated with COVID-19, increasing the chances of death in hospitalized patients [2].

Other fungal infections, particularly those affecting the skin, have emerged as significant concerns. Sporotrichosis, caused by the fungus *Sporothrix* spp., is a mycosis that affects both humans and animals, leading to the development of subcutaneous tissue lesions. This thermally dimorphic fungus exists as a filamentous form in soils rich in organic matter and plant surfaces (25°C) and as a yeast form in the lesions and exudates caused by the disease (37°C) [5].

Cats are the primary vectors in the transmission of sporotrichosis, with the disease being transmitted to both felines and humans predominantly through skin injuries [5]. Commonly, these injuries arise from punctures, bites, or scratches inflicted by domestic cats, or via exposure to their infected secretions. Sporotrichosis typically presents as isolated or multiple lesions; however, in individuals with compromised immune systems, it can progress to a systemic infection, affecting multiple organs [5, 6, 7].

Conventional treatment for infections caused by *Sporothrix* spp. and *Candida* spp. typically involves the administration of oral antifungals such as azole class, potassium iodide, and amphotericin B. Itraconazole is the drug of choice at doses of 100–200 mg/ 24h for humans and 8.3–27.7 mg/kg/24h for cats [5]. However, treatments are usually long, and it is common for patients undergoing therapy to experience numerous side effects, including hepatotoxicity, vomiting, nephrotoxicity, anorexia, and depression [4, 6]. Moreover, reports of drug-resistant strains [6, 8] have led to the exploration of new, safe, effective therapeutic approaches [5] in managing fungal infections.

In this context, Photodynamic Inactivation (PDI) emerges as an alternative to conventional therapy because it has been effective against infections caused by various types of microorganisms [3], particularly those affecting the skin. PDI is a technique that utilizes light of specific wavelengths and a photosensitizing molecule, which, in combination with molecular oxygen, triggers photochemical reactions leading to the formation of reactive oxygen species (ROS). These ROS cause the death of target cells, as pathogenic microorganisms [9]. Different photosensitizers have been used against fungi, such as the ruthenium complex [4], 5-aminolevulinic acid [10], and methylene blue [11].

Curcumin, extracted from the rhizome of *Curcuma longa*, is a molecule that possesses medicinal properties including antioxidant, anti-inflammatory, and antimicrobial properties. It has also photodynamic properties with absorption in the wavelength range of 400 to 500 nm [3, 12] and can be utilized as a photosensitizer for superficial lesions and early-stage treatments. Curcumin has already been used in studies against infections that are unresponsive to conventional treatments, showing promising results in the treatment of mycoses [3, 4, 12] and candidiasis [13, 14]. Curcumin has already been used as an effective photosensitizer in studies against the fungi *Candida albicans* [13, 14] and *Sporothrix* spp. [15].

Due to the ineffectiveness of conventional drugs, resulting in super-long treatments, there has been a growing interest in studying the combination of pharmacological therapy with photodynamic inactivation to increase treatment response [16]. Bacteria and fungi can exhibit higher elimination rates when antimicrobials are used synergistically together with light and a photosensitizer [17]. However, it is important to understand that such therapeutic combinations can also impair the overall therapeutic effect [18]. Therefore, the present study aims to prove the efficacy of Photodynamic Inactivation not only in controlling *Sporothrix brasiliensis*, *Sporothrix schenckii*, and *Candida albicans*, individually but also in reducing the concentrations of traditional antifungals to minimize the side effects caused by them, using curcumin as a photosensitizer, by assessing the combination of PDI with antifungal therapy in *in vitro* tests.

## Methods

### Fungal strains and growth conditions

Yeasts of *S*. *brasiliensis* (CFP 0055) and *S*. *schenckii* (CFP 00746) were cultured in Yeast Extract Peptone Dextrose (YPD) medium at 37°C and pH 7.8 for 10 days. *C*. *albicans* (ATCC 90028) was cultured in Sabouraud Dextrose Agar (SDA) medium at 37°C for 24 hours. Colonies of *S*. *brasiliensis*, *S*. *schenckii*, and *C*. *albicans* were resuspended in 10 mL of saline solution and centrifuged at 4.000 RPM for 10 minutes to discard the supernatant. After repeating this procedure three times, the fungi were resuspended in saline solution at a concentration equivalent to 10^4^−10^5^ CFU/mL.

### Fluorescence Confocal Microscopy (FCM)

To assess the internalization of curcumin by *Sporothrix spp*. and the best time to define the drug-light interval, confocal fluorescence microscopy was performed (LSM 780—Zeiss, Germany) in *S*. *brasiliensis* (CFP 0055) assays. The concentration of curcumin was 5 mg/mL, and the fungal concentration was 10^5^ CFU/mL in the sample. Fluorescence collection occurred in the range of 400 to 700 nm, and images were obtained with excitation at 450 nm. The tested incubation times were 0, 10, 20, 30, 40, 50, and 60 minutes at 25°C.

### Photodynamic Inactivation (PDI)

The photosensitizer used was synthetic curcumin (PDT Pharma, Cravinhos, Brazil), solubilized in 1% DMSO and 99% absolute ethanol to create a stock solution at a concentration of 1.5 mg/mL. The tested concentrations were 0.75, 7.5, 15, 75, and 150 μg/mL, which were obtained after dilution in sterile distilled water.

The tests were conducted in 24-well plates (n = 3). The experimental groups tested were Control (500 μL of inoculum + 500 μL of saline), Light (500 μL of inoculum + 500 μL of saline) under illumination, Only Photosensitizer (500 μL of inoculum + 500 μL of curcumin solution), and PDI (500 μL of inoculum + 500 μL of curcumin solution) under illumination. After an incubation period with the photosensitizer equivalent to 20 minutes in the dark, the Light and PDI groups were illuminated by a homemade device consisting of an array of 24 LEDs emitting light at 450 ±10 nm, irradiance of 35 mW/cm^2^, delivering a fluence of 31.5 J/cm^2^

The contents of each well were then serially diluted to 10^−4^ and plated on SDA solid medium. Colony counting was performed after 24 hours of growth for C. *albicans* and after 72 hours for the *Sporothrix* species.

### Minimal Inhibitory Concentration (MIC)

To determine the lowest concentration capable of reducing the growth of the studied strains, the broth microdilution test was performed to determine the minimum inhibitory concentration according to the Clinical and Laboratory Standard Institute (CLSI) using the medium RPMI 1640 with MOPS. [19].

The antifungals tested were itraconazole (ITZ), ketoconazole (KTZ), and potassium iodide (KI) at concentrations of 16, 8, 4, 2, 1, 0.5, 0.25, 0.125, 0.0625, and 0.0312 μg/mL for ITZ and KTZ, and 250, 125, 62.5, 31.25, 15.62, 7.8, 3.9, 1.95, 0.97, and 0.488 mg/mL for KI.

Initially, *C*. *albicans*, *S*. *brasiliensis*, and *S*. *schenckii* yeasts were cultured in SDA medium at 37°C for 24 hours for *C*. *albicans* and 10 days for *S*. *schenckii* and *S*. *brasiliensis*. After this period, the optical density was adjusted to a concentration of 10^4^−10^5^ CFU/mL for all three species at 530 nm. The antifungals were added to the microdilution plate, and they were incubated at 37°C for 24 hours for *C*. *albicans* and 72 hours for *S*. *brasiliensis* and *S*. *schenckii* to determine the MIC. The MIC was defined as the lowest concentration of the antifungal agent at which no visible fungal growth was observed, indicated by the last clear well before any turbidity appeared. The experiment was done in triplicate and the value of MIC was obtained by calculating the mode.

### Association of therapies

For the combination of therapies, subinhibitory values of both PDI and antifungals were used. Subinhibitory concentrations of curcumin were used for PDT, which caused partial microbial death (reduction of 2 to 3 logs). For the antifungals, half or less of the determined MIC value for each drug was used for each microorganism. Thus, the best reductions result for individual therapies at subinhibitory doses and the combination with PDI applied before and after antifungal therapy were analyzed, in triplicate. The optical density of the inoculum was adjusted to an initial fungal concentration of 10^4^−10^5^ CFU/mL for the microorganisms.

The tests were divided into groups based on the treatment. The Control group did not receive any treatment. The PDI group received treatment with underdoses of curcumin, using concentrations of 7.5 μg/mL of curcumin for *C*. *albicans* and *S*. *brasiliensis*, and 0.75 μg/mL for *S*. *schenckii*, DLI of 20 minutes and light dose of 31.5 J/cm^2^. The Antifungal monotherapy (Drug underdosing group) received treatment only with the antifungal solution and was plated after 24-hour interaction with the drug. The drug+PDI group received antifungal underdose treatment first, and, after 24 hours of interaction, was followed by PDI in the subinhibitory concentration. The PDI+drug group received PDI underdose treatment first, and then, the antifungal subinhibitory dose of treatment, being plated 24 hours later.

All groups were plated on SDA medium in 33 μL aliquots at dilutions of 10^0^, 10^−1^, and 10^−2^, and the plates were counted after colony growth, which varied from 24 hours for *C*. *albicans* to 72 hours for *S*. *brasiliensis* and *S*. *schenckii*.

### Statistical analysis

The experiments were performed in triplicates for each group studied and the results were analyzed by ANOVA and Tukey test. The p-value < 0.05 was considered statistically significant when compared to the control group.

## Results

### Only Photodynamic Inactivation

#### Determination of Drug-Light Interval (DLI)

**Fig 1:**
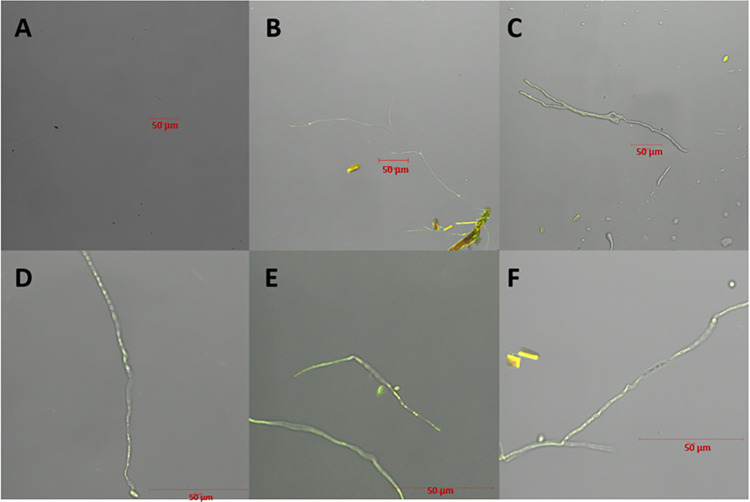
The images obtained with Confocal Fluorescence Microscopy demonstrate the affinity of curcumin with *S*. *brasiliensis* at incubation times of 0 minutes (A), 10 minutes (B), and 20 minutes (C), where there is effective internalization of the photosensitizer into the fungus, 30 minutes (D), 40 minutes (E), and 60 minutes (F).

The obtained images from the confocal microscope show that the process of total curcumin internalization in *S*. *brasiliensis* begins after 20 minutes of incubation (Fig 1), at which point there was fluorescence emission within the fungal cell wall. Although the photosensitizer remained within the microorganism, the fluorescence emission decreased in 60 min. Therefore, the drug-light interval was defined as 20 minutes for the PDT tests.

#### PDI groups

**Fig 2:**
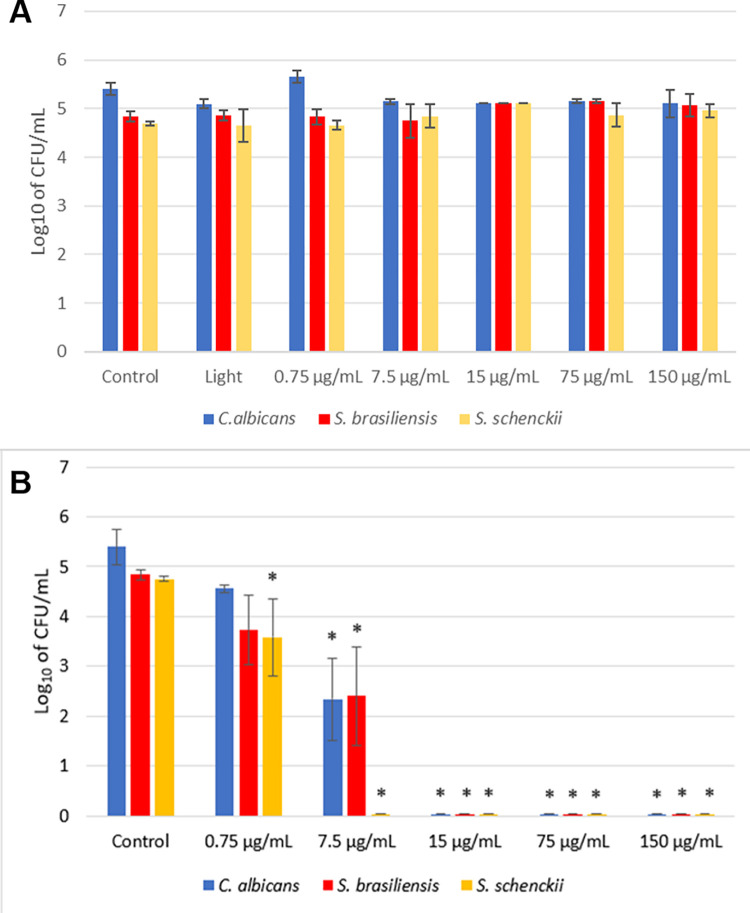
A. Toxicity tests with *C*. *albicans*, *S*. *brasiliensis*, and *S*. *schenckii*, when light at fluence of 31.5 J/cm^2^ or different curcumin concentrations are applied. *indicates statistical significance when compared to the control group (p < 0.05) (n = 3). B. Photodynamic inactivation of *C*. *albicans*, *S*. *brasiliensis*, and *S*. *schenckii*, with different curcumin concentrations and light fluence of 31.5 J/cm^2^. *indicates statistical significance when compared to the control group (p < 0.05) (n = 3).

The “Control”, “Light”, and “Only Photosensitizer” groups were tested to assess the toxicity of each element, individually. Even the highest concentration of the photosensitizer tested was not able to reduce the population of any of the microorganisms in the absence of light, as well as the Only Light group, with 31.5 J/cm^2^ at 35 mW/cm^2^ (Fig 2A), indicating that, individually, both the light and PS elements do not have a microbicidal effect.

When the elements were combined in the photodynamic process, different concentrations of curcumin were tested for the three microorganisms with the same light doses. In relation to *C*. *albicans*, there was a reduction of 3.06 log_10_ with a curcumin concentration of 7.5 μg/mL and complete inactivation of the fungus with a concentration from 15 μg/mL of the same photosensitizer. In the same way, *S*. *brasiliensis* showed a reduction of 2.44 log_10_ CFU/mL with a concentration of 7.5 μg/mL and complete inactivation from 15 μg/mL. Regarding *S*. *schenckii*, there was a reduction of 1.17 log_10_ with 0.75 μg/mL of curcumin and complete inactivation with 7.5 μg/mL, half the concentration needed to completely inactivate *S*. *brasiliensis*. The results for all concentrations and each microorganism are shown in Fig 2B.

### Only antifungal–Minimal Inhibitory Concentration (MIC)

The MIC values for the antifungals ITZ, KTZ, and KI were determined based on the mode calculation for the microorganisms *C*. *albicans*, *S*. *brasiliensis*, and *S*. *schenckii*. The values are shown in Table 1, with the highest concentration of ITZ (2 μg/mL) required for the inactivation of *S*. *brasiliensis*, the highest concentrations of KTZ (0.25 μg/mL) for *C*. *albicans* and *S*. *brasiliensis*, and the highest concentration of KI also for *S*. *brasiliensis*, at 250 mg/mL.

**Table 1:** MIC values found in the fungal susceptibility test.

Microorganism	Drug		
	ITZ (μg/mL)	KTZ (μg/mL)	KI (mg/mL)
*C*. *albicans*	0.125	0.250	31.25
*S*. *brasiliensis*	2.0	0.250	250
*S*. *schenckii*	0.250	0.125	62.5

### Association of therapies

After conducting PDI and MIC tests individually against all the fungi studied, the best parameters were analyzed and used in combination, with subinhibitory concentrations of both PDI and antifungals changing the order of application.

#### Candida albicans

For *C*. *albicans*, when ITZ was used as monotherapy (Drug underdosing group) at a concentration of 0.0625 μg/mL, a reduction of 0.9 log_10_ was observed compared to the control group. With a concentration of 7.5 μg/mL of curcumin, only PDI showed a reduction of 1.72 log_10_. When the antifungal was used before PDI (Drug+PDI group), the reduction was 2 log_10_, indicating an effectiveness of only 1.1 log_10_ when compared to using only the antifungal and an insignificant reduction compared to the effect of the PDI group, showing that there was no additive effect. However, in the group where PDI was performed before the application of the ITZ (PDI+drug group), the combination therapy resulted in a synergistic effect, with a reduction of 5.2 logs_10_ in the *C*. *albicans* population compared to the control group.

Analyzing the effect of KTZ alone, it caused a reduction of 0.86 log_10_ in CFU/mL at a concentration of 0.125 μg/mL, and a reduction of 1.97 log_10_ for the Drug+PDI group. When PDI was applied before KTZ, there was a reduction of 5.2 logs_10_ in the *C*. *albicans* population, showing a synergistic effect again. Also, with up to 25% of the concentration obtained in the MIC of KTZ, that is, 0.0625 μg/mL, it was possible to reduce the *C*. *albicans* population by 4.67 log_10_ in the PDI+drug group.

**Fig 3:**
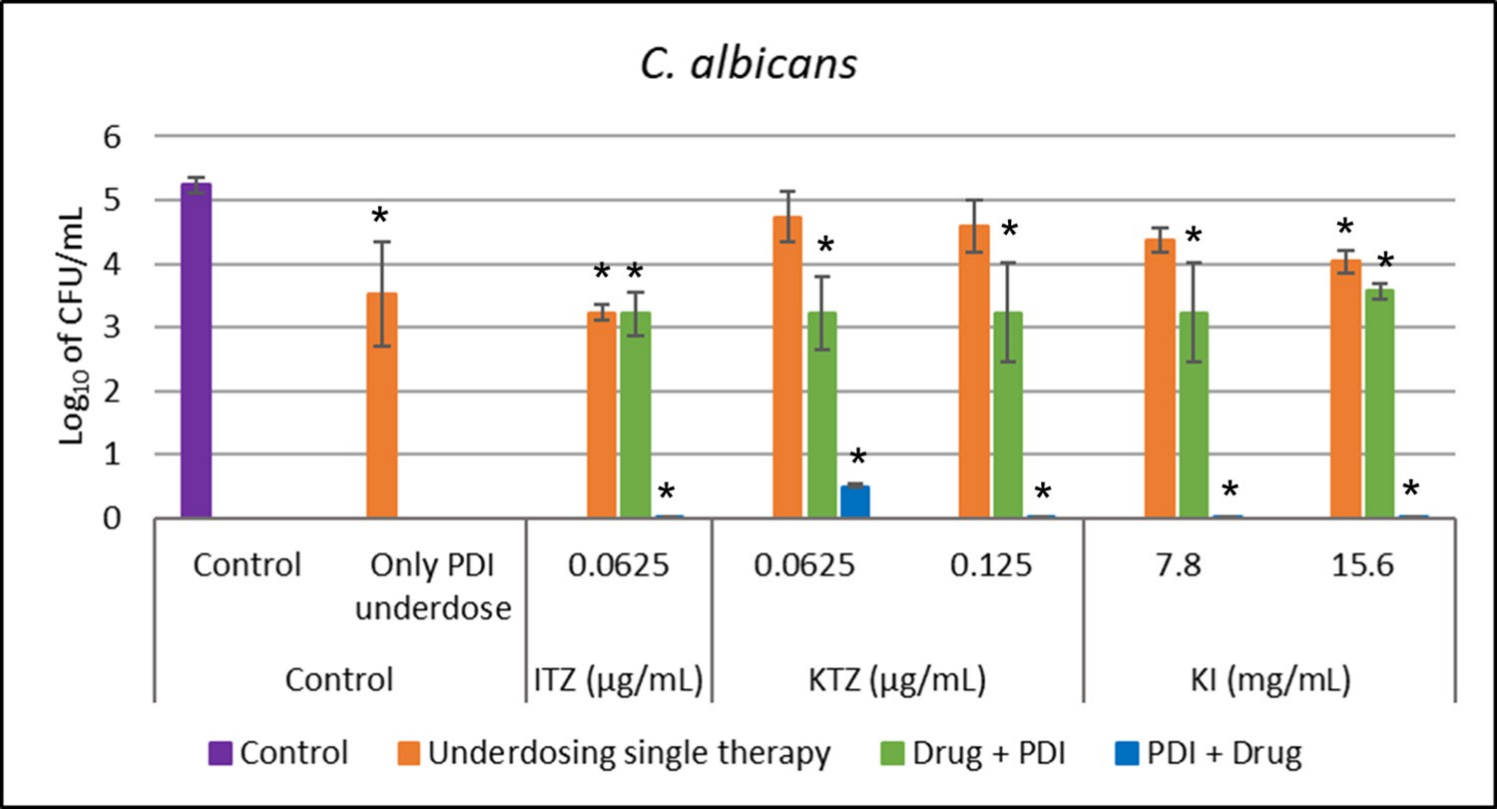
*In vitro* test for all conditions against *C*. *albicans*, in comparison with a control group (without any treatment). Underdosing single therapies (orange bar) like Only PDI with 7.5 ug/mL of curcumin and 31.5 J/cm2 and all antifungals (ITZ, KTZ, and KI) in different concentrations (orange bar). The combination of both therapies was evaluated when PDI was applied after (Drug+PDI–green bar) and before (PDI+Drug–blue bar) each antifungal. *indicates statistical significance when compared to the control group (p < 0.05) (n = 3).

KI as monotherapy reduced 1.4 log_10_ of CFU/mL at a concentration of 15.6 mg/mL. When combined with PDI (Drug+PDI group), a reduction of 1.6 log_10_ was observed. A reduction of 3.9 log_10_ with a KI concentration of 7.8 mg/mL was shown when PDI was performed before the application of this antifungal until complete reduction of this fungus was observed with a concentration of 15.6 mg/mL (PDI+drug group). These data are shown in Fig 3.

#### Sporothrix brasiliensis

For *S*. *brasiliensis*, the underdose concentration of PDI used was 7.5 μg/mL of curcumin, which resulted in a reduction of 2.33 CFU/mL. ITZ at 1 μg/mL alone reduced 2.81 log_10_ CFU/mL. The combination of therapies caused a reduction of 1.4 log_10_ in the Drug+PDI group, meaning less than the antifungal alone, and a complete reduction compared to the control (4.8 CFU/mL) in the PDI+drug group. When PDI was applied before, it was possible to reduce the fungus by 4.8 log10 at an ITZ concentration of 0.125 μg/mL, meaning the combination in the correct order was able to completely inactivate *S*. *brasiliensis*, even with concentrations up to 16 times lower than the MIC of itraconazole.

KTZ alone reduced 0.5 log_10_ at a concentration of 0.125 μg/mL. The Drug+PDI combination caused a reduction of 2.8 log_10_, and when PDI was applied before (PDI+drug group), there was a complete reduction compared to the control (4.8 CFU/mL) and reached a reduction of 3.04 log_10_ with KTZ concentrations up to 25% lower than MIC.

**Fig 4:**
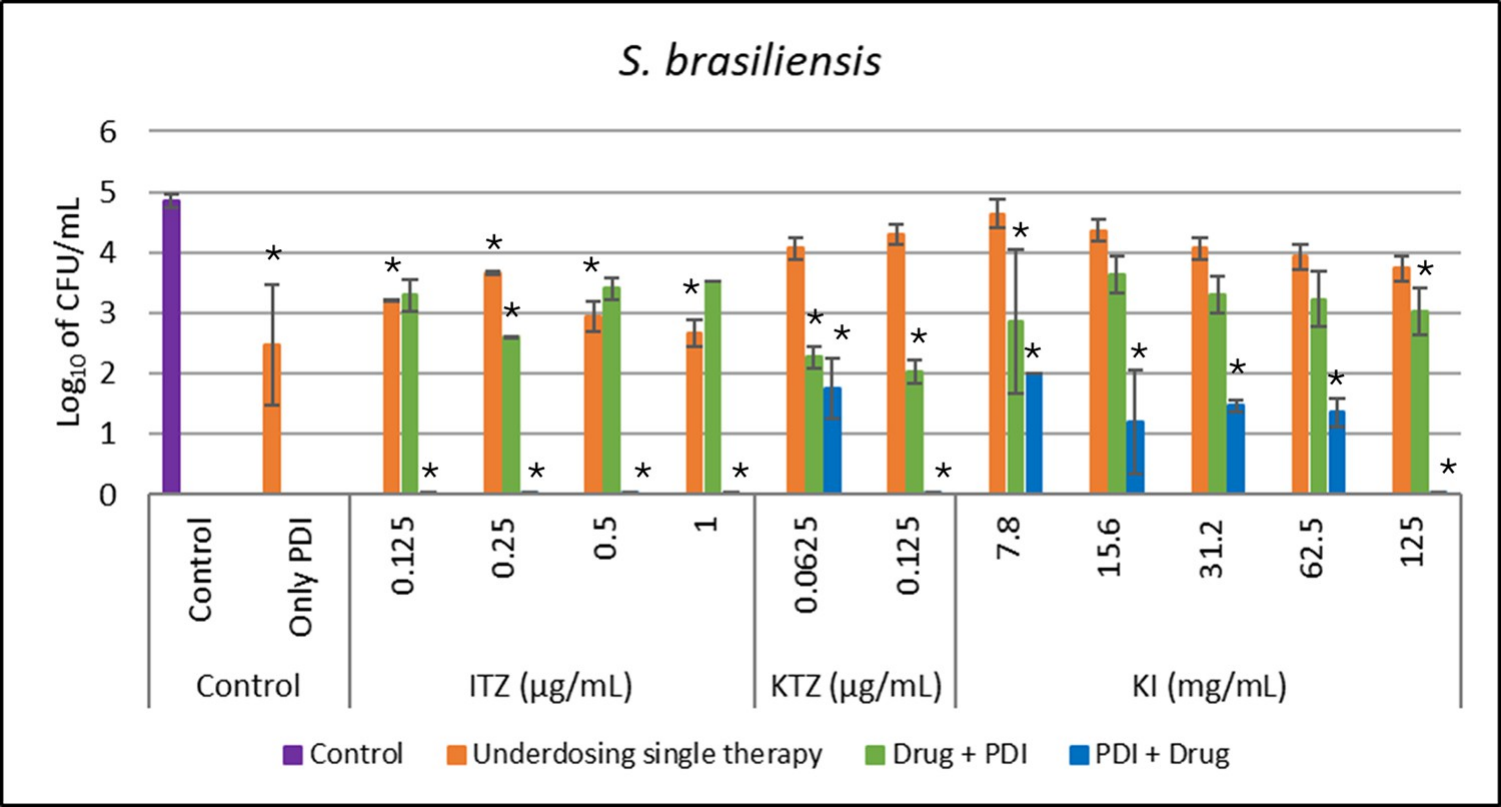
*In vitro* test for all conditions against *S*. *brasiliensis*, in comparison with a control group (without any treatment). Underdosing single therapies (orange bar) like Only PDI with 7.5 ug/mL of curcumin and 31.5 J/cm2 and all antifungals (ITZ, KTZ, and KI) in different concentrations (orange bar). The combination of both therapies was evaluated when PDI was applied after (Drug+PDI–green bar) and before (PDI+Drug–blue bar) each antifungal. *indicates statistical significance when compared to the control group (p < 0.05) (n = 3).

For KI, when applied alone, there was a reduction of 1.1 log_10_ in the microbial population compared to the control group at a concentration of 125 mg/mL. The Drug+PDI group reduced 1.8 log_10_, and the PDI+drug group caused complete elimination compared to the control (4.8 log10 CFU/mL) of *S*. *brasiliensis*, showing a synergistic effect in the PDI+drug group for all antifungals. All these groups are shown are shown in Fig 4.

#### Sporothrix schenckii

All underdoses antifungals applied as monotherapy to *S*. *schenckii* caused a reduction of up to 1.1 log_10_ CFU/mL in this microorganism, showing a non-significant reduction compared to the control group. The underdose of PDI was applied at a concentration of 0.75 μg/mL of curcumin, resulting in a reduction of 3.5 log_10_ CFU/mL when applied alone.

**Fig 5:**
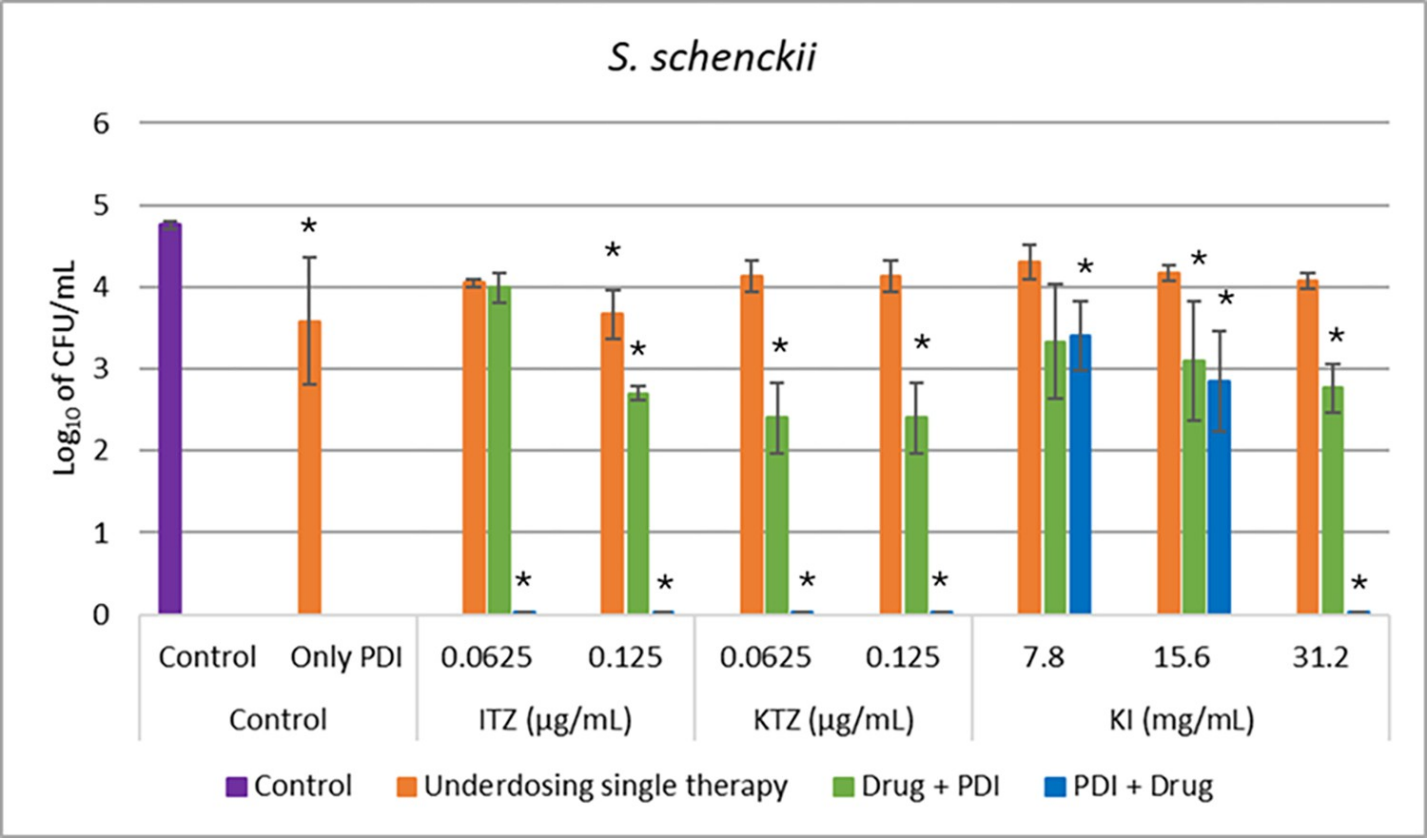
*In vitro* test for all conditions against *S*. *schenckii*, in comparison with a control group (without any treatment). Underdosing single therapies (orange bar) like Only PDI (orange bar) with 0.75 ug/mL of curcumin and 31.5 J/cm2 and all antifungals (ITZ, KTZ, and KI) in different concentrations (orange bar). The combination of both therapies was evaluated when PDI was applied after (Drug+PDI–green bar) and before (PDI+Drug–blue bar) each antifungal. *indicates statistical significance when compared to the control group (p < 0.05) (n = 3).

The Drug+PDI combination caused a reduction of 1.9 log_10_ at a concentration of 0.125 μg/mL of ITZ, 2.3 log_10_ at a concentration of 0.065 μg/mL of KTZ, and 1.9 log_10_ with 31.2 mg/mL of KI. The PDI+drug group caused a complete reduction in *S*. *schenckii* compared to the control (4.7 log_10_ CFU/mL) with the use of the three antifungals tested, showing synergy in this group, even with the ITZ concentration four times lower than the MIC value for this drug. The data for all combinations for this microorganism are shown in Fig 5.

**Fig 6:**
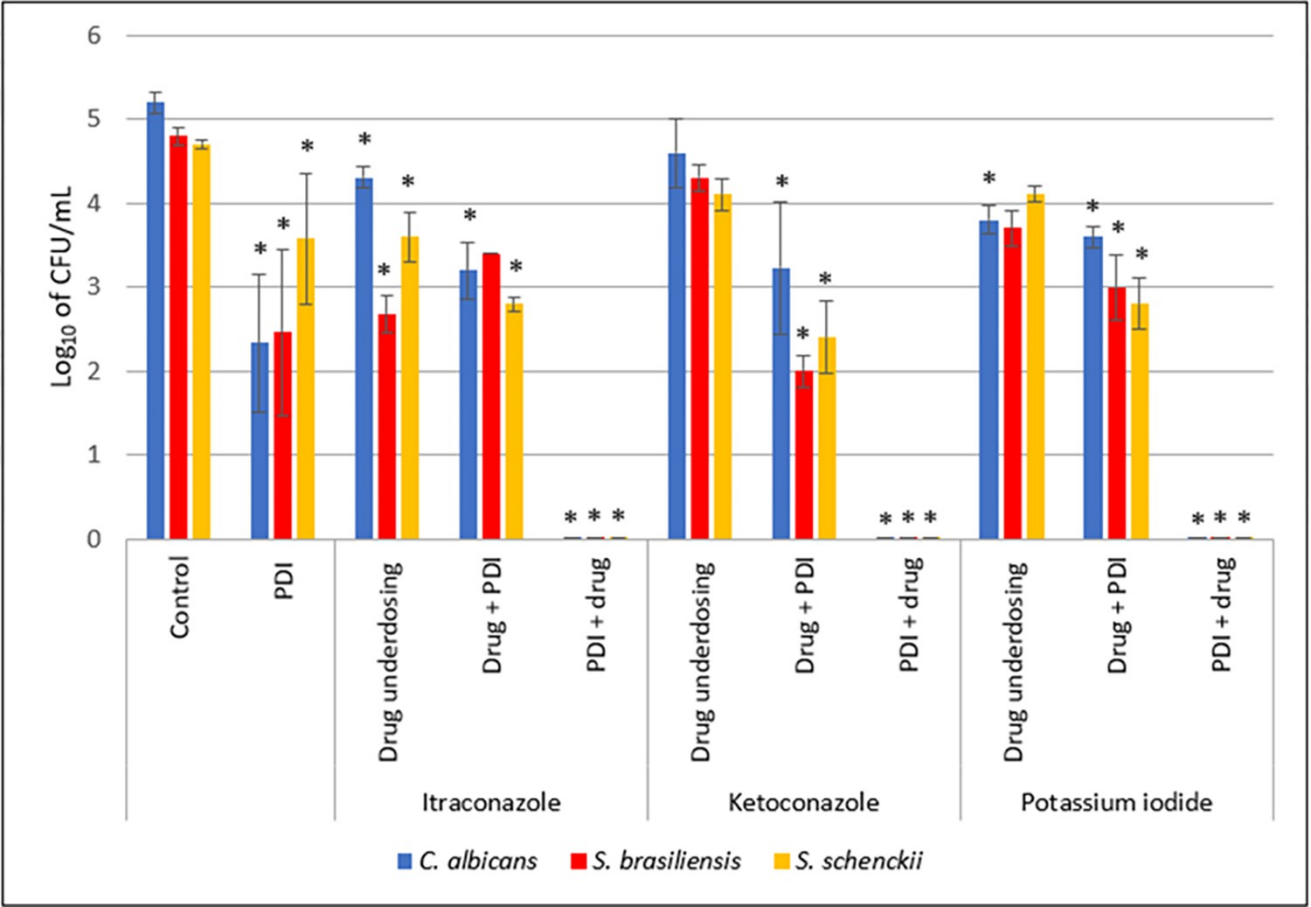
Evaluation of *C*. *albicans*, *S*. *brasiliensis*, and *S*. *schenckii* in the presence of a combination of PDI (7.5 μg/mL of curcumin to *C*. *albicans* and *S*. *brasiliensis* and 0.75 μg/mL of curcumin to *S*. *schenckii*) with concentrations equivalent to half the MIC value of antifungals itraconazole (0.0625 μg/mL to *C*. *albicans*, 1μg/mL to *S*. *brasiliensis* and 0.125 μg/mL to *S*. *schenckii*), ketoconazole (0.125 μg/mL to *C*. *albicans* and *S*. *brasiliensis* and 0.0625 μg/mL to *S*. *schenckii*), and potassium iodide (15.625 mg/mL to *C*. *albicans*, 125 mg/mL to *S*. *brasiliensis* and 31.25 mg/mL to *S*. *schenckii*) in different orders of application. *indicates statistical significance when compared to the control group (p < 0.05) (n = 3).

To compare all microorganisms and the importance of the application order of each therapy, a graph with the subinhibitory doses of PDI and the three drugs individually and when PDI was applied before and after antifungal was shown for half the MIC concentration in Fig 6. This graph makes it clear that when PDI is applied first, there is always a synergistic effect of the combined therapies.

## Discussion

Species such as the *Sporothrix* complex produce melanin as a virulence factor, providing these microorganisms with protection against antifungal drugs and reducing the efficacy of medications used to treat sporotrichosis [18]. On the other hand, cases of resistance in *Candida* species are often related to delays in diagnosis and consequently a delay in the effective treatment of the disease [2]. Therefore, the development of other therapeutic modalities that can effectively and quickly treat these infections is necessary.

Candidiasis and sporotrichosis are already being addressed using PDI. In *in vitro* studies evaluating the photodynamic activity of curcumin against *Candida* species achieved a 7 log_10_ reduction in CFU/mL of *C*. *albicans* suspensions using natural curcumin at a concentration equivalent to 7.4 μg/mL, and a fluence of 5.28 J/cm^2^ [13]. Our study achieved complete inactivation of *C*. *albicans* with a concentration of 15 μg/mL of synthetic curcumin, and 31.5 J/cm^2^. This difference may be attributed to the type of curcumin used. In a study comparing different curcumin formulations, greater reductions in bacterial populations were obtained using natural curcumin compared to synthetic ones [20]. Nevertheless, both curcumins show promising results against *C*. *albicans*, making them a promising therapy against this fungus.

In our study, the higher concentration of curcumin used to inactivate *S*. *brasiliensis* compared to *S*. *schenckii* can be explained by *S*. *brasiliensis* has a thicker cell wall and longer outer microfibrils.[8] It is also known that S. *brasiliensis* is a more virulent fungus, causing more severe and disseminated lesions in infections and being more strongly associated with mouse mortality than *S*. *schenckii* [7].

The great results obtained with only PDI are due to the ability of curcumin to penetrate and interact with the fungal cell membrane. Although various photosensitizers have been employed in the photodynamic inactivation of *Sporothrix* spp. [4,10,11,21], none, except for the ruthenium complex, demonstrated a superior reduction in fungal activity compared to curcumin.

Additionally, curcumin is a cost-effective option with commercial availability for potential clinical treatments. Another molecule with similar characteristics is methylene blue. However, it is a contraindicated compound for cats, causing oxidative damage in red blood cells in the primary transmitters of this disease, making effective sporotrichosis treatment unfeasible [22].

A recent study confirmed the *in vitro* antimicrobial activity of curcumin against *S*. *brasiliensis*, completely inactivating both filamentous and yeast-like forms of this fungus [15]. However, our study showed the combination of PDI and traditional antifungals. When the combination of therapies is done in a controlled manner, it is possible to reduce the quantity of traditional antifungals and, consequently, may reduce the undesirable effects caused by their prolonged use in clinical use. [17]

The superior results obtained when PDI was performed before antifungal treatment can be attributed to the damage to the microbial cell membrane. [11] This alteration in cell permeability facilitates the entry of the drug into these microorganisms, consequently leading to increased mortality. [16]

Lan *et al*. analyzed the effects of PDI in association with oral antifungal use and observed the nearly complete inactivation of *Trichophoron asahii* strains in biofilm assays of this species using ALA as a photosensitizer, showing better results when PDI was performed before [23]. The authors also attributed the breakage of fungal resistance to morphological changes in the microorganism caused by PDI, which reinforces that applying PDI first not only enhances the effect of drugs by increasing the permeability of the microorganism’s cell membrane, thus facilitating the internalization of these drugs, and contributing to their greater efficacy, but it can also alter the mechanism of resistance to them with metabolic changes [9, 24].

The reduction in effectiveness when PDI is applied after drug treatment can be explained by the presence of other molecules that act as a trap of singlet oxygen, making less of this molecule available in the system for microbial death compared to PDI alone [9, 25]. Therefore, there may be a reduction in the photodynamic effect when the antifungal is used before the photosensitizer with the change of photochemistry properties of PS.

A suggested hypothesis, which could explain the greater photoinactivation when both therapies are combined, is related to the fact that the antifungal weakens the cell wall of the microorganism, through the reduction of ergosterol synthesis by azoles (itraconazole and ketoconazole) [26], causing greater penetration of the photosensitizer in the cell. However, subinhibitory doses would not be capable of causing serious damage to the cell wall and, in addition, the photodynamic effect can destroy the antifungal molecule. Despite studies demonstrating the synergy of KI with PDI in the inactivation of bacteria [27] and fungi [28], our study observed that the association of this molecule causes a similar or even reduced death effect when compared to isolated drug therapies and photodynamic inactivation. These differences may be explained by variations in the experimental conditions such as the photosensitizer used and the sequence of therapies, as well as its combination with other techniques [27,28].

Understanding these processes helps us plan the best treatment protocol, whether for the use of PDI as monotherapy or in combination with pharmacological therapy, as well as the order of application. This seeks to provide effective and safe therapeutic solutions, leading to better treatment adherence, reduced time to cure, lower costs, fewer side effects, and reduced microbial resistance. Therefore, Photodynamic Inactivation using curcumin as a photosensitizer emerges as a promising alternative to conventional treatment for sporotrichosis and infections caused by *Candida albicans* and *Sporothrix* spp. Despite its limited penetration into deeper biological tissues, its combination with antifungals can significantly enhance treatment response by reducing the need for high concentrations of antifungals thereby positively impacting treatment duration, costs, and side effects. Nevertheless, the sequence of combining antifungal and PDI can influence efficacy and outcomes in microbial elimination, with notably synergistic outcomes observed when PDI precedes antifungal administration.

## Supporting information

S1 Table Data related to Fig 2A. (DOCX)

S2 Table Data related to Fig 2B. (DOCX)

S3 Table Data related to Fig 3. (DOCX)
