# Supplementary material for: Photodynamic inactivation strategies for maximizing antifungal effect against Sporothrix spp. and Candida albicans in an in vitro investigation
Source: PLoS Negl Trop Dis. 2024 Nov 12;18(11):e0012637. doi: 10.1371/journal.pntd.0012637 (PMC11594586; doi:10.1371/journal.pntd.0012637)
Supplement: S1 Table — (DOCX) [file pntd.0012637.s001.docx]

**Supporting Information for**

Photodynamic Inactivation Strategies for Maximizing Antifungal Effect Against *Sporothrix* spp. and *Candida albicans* in an *In Vitro* Investigation

*Amanda Regina Rocha^1,2^, Natalia Mayumi Inada^2^, Ana Paula da Silva^2^, Vanderlei Salvador Bagnato^1,2,3^, Hilde Harb Buzzá^4*^*

^1^PPG Biotec, Federal University of São Carlos, São Carlos, Brazil

^2^ São Carlos Institute of Physics, University of São Paulo, São Carlos, Brazil

^3^ Department of Biomedical Engineering, Texas A&M University, College Station, USA

^4^ Institute of Physics, Pontificia Universidad Catolica de Chile, Santiago, Chile.

Corresponding author: [hilde.buzza@uc.cl](mailto:hilde.buzza@uc.cl)

Table S1 – Data related to Figure 2A

| *C. albicans* | | | |
| --- | --- | --- | --- |
| **Group** | **CFU/mL** | **Average** | **SD** |
| Control | 5.1 | 5.233333 | 0.124722 |
|  | 5.2 |  |  |
|  | 5.4 |  |  |
| Light | 5 | 5.106667 | 0.089938 |
|  | 5.22 |  |  |
|  | 5.1 |  |  |
| 150 μg/mL | 5.4 | 5.143333 | 0.236126 |
|  | 4.83 |  |  |
|  | 5.2 |  |  |
| 75 μg/mL | 5.2 | 5.17 | 0.042426 |
|  | 5.11 |  |  |
|  | 5.2 |  |  |
| 15 μg/mL | 5.36 | 5.523333 | 0.139124 |
|  | 5.7 |  |  |
|  | 5.51 |  |  |
| 7,5 μg/mL | 5.21 | 5.146667 | 0.046428 |
|  | 5.1 |  |  |
|  | 5.13 |  |  |
| 0,75 μg/mL | 5.52 | 5.35 | 0.125698 |
|  | 5.22 |  |  |
|  | 5.31 |  |  |

| *S. brasiliensis* | | | |
| --- | --- | --- | --- |
| **Group** | **CFU/mL** | **Average** | **SD** |
| Control | 4.91 | 4.846667 | 0.10403 |
|  | 4.93 |  |  |
|  | 4.7 |  |  |
| Light | 5 | 4.863333 | 0.097411 |
|  | 4.78 |  |  |
|  | 4.81 |  |  |
| 150 μg/mL | 5.3 | 5.076667 | 0.192585 |
|  | 4.83 |  |  |
|  | 5.1 |  |  |
| 75 μg/mL | 5.2 | 5.153333 | 0.036818 |
|  | 5.11 |  |  |
|  | 5.15 |  |  |
| 15 μg/mL | 4.87 | 4.933333 | 0.053125 |
|  | 5 |  |  |
|  | 4.93 |  |  |
| 7,5 μg/mL | 4,4 | 4,7 | 0.294392 |
|  | 5,1 |  |  |
|  | 4,6 |  |  |
| 0,75 μg/mL | 4,61 | 4,836667 | 0,165395 |
|  | 5 |  |  |
|  | 4,9 |  |  |

| *S. schenckii* | | | |
| --- | --- | --- | --- |
| **Group** | **CFU/mL** | **Average** | **SD** |
| Control | 4,74 | 4,79 | 0,037417 |
|  | 4,83 |  |  |
|  | 4,8 |  |  |
| Light | 4.56 | 4.653333 | 0,3332 |
|  | 4.3 |  |  |
|  | 5.1 |  |  |
| 150 μg/mL | 5.1 | 4.977667 | 0.111673 |
|  | 4.83 |  |  |
|  | 5.003 |  |  |
| 75 μg/mL | 4.64 | 4.883333 | 0.192238 |
|  | 5.11 |  |  |
|  | 4.9 |  |  |
| 15 μg/mL | 4.36 | 4.53 | 0.138804 |
|  | 4.7 |  |  |
|  | 4.53 |  |  |
| 7,5 μg/mL | 5.1 | 4.7 | 0.294392 |
|  | 4.6 |  |  |
|  | 4.4 |  |  |
| 0,75 μg/mL | 4.52 | 4.66 | 0.10198 |
|  | 4.76 |  |  |
|  | 4.7 |  |  |
