# Supplementary material for: Photodynamic inactivation strategies for maximizing antifungal effect against Sporothrix spp. and Candida albicans in an in vitro investigation
Source: PLoS Negl Trop Dis. 2024 Nov 12;18(11):e0012637. doi: 10.1371/journal.pntd.0012637 (PMC11594586; doi:10.1371/journal.pntd.0012637)
Supplement: S2 Table — (DOCX) [file pntd.0012637.s002.docx]

**Supporting Information for**

Photodynamic Inactivation Strategies for Maximizing Antifungal Effect Against *Sporothrix* spp. and *Candida albicans* in an *In Vitro* Investigation

*Amanda Regina Rocha^1,2^, Natalia Mayumi Inada^2^, Ana Paula da Silva^2^, Vanderlei Salvador Bagnato^1,2,3^, Hilde Harb Buzzá^4*^*

^1^PPG Biotec, Federal University of São Carlos, São Carlos, Brazil

^2^ São Carlos Institute of Physics, University of São Paulo, São Carlos, Brazil

^3^ Department of Biomedical Engineering, Texas A&M University, College Station, USA

^4^ Institute of Physics, Pontificia Universidad Catolica de Chile, Santiago, Chile.

Corresponding author: [hilde.buzza@uc.cl](mailto:hilde.buzza@uc.cl)

Table S2 - Data related to Figure 2B.

| *C. albicans* | | | |
| --- | --- | --- | --- |
| **Group** | **CFU/mL** | **Average** | **SD** |
| Control | 5.1 | 5.4 | 0.355903 |
|  | 5.2 |  |  |
|  | 5.9 |  |  |
| 150 μg/mL | 0 | 0 | 0 |
|  | 0 |  |  |
|  | 0 |  |  |
| 75 μg/mL | 0 | 0 | 0 |
|  | 0 |  |  |
|  | 0 |  |  |
| 15 μg/mL | 0 | 0 | 0 |
|  | 0 |  |  |
|  | 0 |  |  |
| 7,5 μg/mL | 2.69 | 2.34 | 1.784956 |
|  | 4.33 |  |  |
|  | 0 |  |  |
| 0,75 μg/mL | 4.64 | 4.546667 | 0.067987 |
|  | 4.48 |  |  |
|  | 4.52 |  |  |

| *S. brasiliensis* | | | |
| --- | --- | --- | --- |
| **Group** | **CFU/mL** | **Average** | **SD** |
| Control | 4.91 | 4.846667 | 0.10403 |
|  | 4.93 |  |  |
|  | 4.7 |  |  |
| 150 μg/mL | 0 | 0 | 0 |
|  | 0 |  |  |
|  | 0 |  |  |
| 75 μg/mL | 0 | 0 | 0 |
|  | 0 |  |  |
|  | 0 |  |  |
| 15 μg/mL | 0 | 0 | 0 |
|  | 0 |  |  |
|  | 0 |  |  |
| 7,5 μg/mL | 2.1 | 2.466667 | 0.98986 |
|  | 1.48 |  |  |
|  | 3.82 |  |  |
| 0,75 μg/mL | 4.1 | 3.733333 | 0.702772 |
|  | 2.75 |  |  |
|  | 4.35 |  |  |

| *S. schenckii* | | | |
| --- | --- | --- | --- |
| **Group** | **CFU/mL** | **Average** | **SD** |
| Control | 4.74 | 4.756667 | 0.054365 |
|  | 4.83 |  |  |
|  | 4.7 |  |  |
| 150 μg/mL | 0 | 0 | 0 |
|  | 0 |  |  |
|  | 0 |  |  |
| 75 μg/mL | 0 | 0 | 0 |
|  | 0 |  |  |
|  | 0 |  |  |
| 15 μg/mL | 0 | 0 | 0 |
|  | 0 |  |  |
|  | 0 |  |  |
| 7,5 μg/mL | 0 | 0 | 0 |
|  | 0 |  |  |
|  | 0 |  |  |
| 0,75 μg/mL | 2.8 | 3.646667 | 0.643808 |
|  | 4.36 |  |  |
|  | 3.78 |  |  |
