# Supplementary material for: Photodynamic inactivation strategies for maximizing antifungal effect against Sporothrix spp. and Candida albicans in an in vitro investigation
Source: PLoS Negl Trop Dis. 2024 Nov 12;18(11):e0012637. doi: 10.1371/journal.pntd.0012637 (PMC11594586; doi:10.1371/journal.pntd.0012637)
Supplement: S3 Table — (DOCX) [file pntd.0012637.s003.docx]

**Supporting Information for**

Photodynamic Inactivation Strategies for Maximizing Antifungal Effect Against *Sporothrix* spp. and *Candida albicans* in an *In Vitro* Investigation

*Amanda Regina Rocha^1,2^, Natalia Mayumi Inada^2^, Ana Paula da Silva^2^, Vanderlei Salvador Bagnato^1,2,3^, Hilde Harb Buzzá^4*^*

^1^PPG Biotec, Federal University of São Carlos, São Carlos, Brazil

^2^ São Carlos Institute of Physics, University of São Paulo, São Carlos, Brazil

^3^ Department of Biomedical Engineering, Texas A&M University, College Station, USA

^4^ Institute of Physics, Pontificia Universidad Catolica de Chile, Santiago, Chile.

Corresponding author: [hilde.buzza@uc.cl](mailto:hilde.buzza@uc.cl)

Table 3 - Data related to Figure 3.

| *C. albicans* | | | | | | |
| --- | --- | --- | --- | --- | --- | --- |
| Itraconazole | CFU/mL | | | | Average | SD |
|  | 0.0625 μg/mL | | | | | |
|  | Drug | 3.35 | 3.1 | 3.25 | 3.2333 | 0.1027 |
|  | Drug + PDI | 3.55 | 2.87 | 3.3 | 3.24 | 0.280832 |
|  | PDI + Drug | 0 | 0 | 0 | 0 | 0 |
| Ketoconazole | 0.0625 μg/mL | | | | | |
|  | Drug | 4.4 | 5.3 | 4.5 | 4.5 | 0.402768 |
|  | Drug + PDI | 3.55 | 2.4 | 3.7 | 3.7 | 0.580708 |
|  | PDI + Drug | 0.53 | 0.53 | 1.4 | 1.4 | 0.410122 |
|  | 0.125 μg/mL | | | | | |
|  | Drug | 4.6 | 5.1 | 4.1 | 4.6 | 0.408248 |
|  | Drug + PDI | 4.1 | 2.2 | 3.4 | 3.233333 | 0.784573 |
|  | PDI + Drug | 0 | 0 | 0 | 0 | 0 |
| Potassium iodide | 7.8 mg/mL | | | | | |
|  | Drug | 4.5 | 4.5 | 4.1 | 4.366667 | 0.188562 |
|  | Drug + PDI | 3.7 | 3.7 | 4 | 3.8 | 0.141421 |
|  | PDI + Drug | 1,3 | 1.3 | 1.3 | 1.3 | 0 |
|  | 15.6 mg/mL | | | | | |
|  | Drug | 4.1 | 4.2 | 3.8 | 4.033333 | 0.169967 |
|  | Drug + PDI | 3.4 | 3.7 | 3.6 | 3.566667 | 0.124722 |
|  | PDI + Drug | 0 | 0 | 0 | 0 | 0 |

| *S. brasiliensis* | | | | | | |
| --- | --- | --- | --- | --- | --- | --- |
| Itraconazole | CFU/mL | | | | Average | SD |
|  | 0.125 μg/mL | | | | | |
|  | Drug | 3.22 | 3.19 | 3.15 | 3.186667 | 0.015 |
|  | Drug + PDI | 3.55 | 3.04 | 3.61 | 3.4 | 0.255 |
|  | PDI + Drug | 0 | 0 | 0 | 0 | 0 |
|  | 0.250 μg/mL | | | | | |
|  | Drug | 3.68 | 3.64 | 3.82 | 3.713333 | 0.02 |
|  | Drug + PDI | 2.61 | 2.6 | 2.8 | 2.67 | 0.005 |
|  | PDI + Drug | 0 | 0 | 0 | 0 | 0 |
|  | 0.5 μg/mL | | | | | |
|  | Drug | 3.2 | 2.7 | 2.91 | 2.936667 | 0.25 |
|  | Drug + PDI | 3.59 | 3.22 | 3.49 | 3.433333 | 0.185 |
|  | PDI + Drug | 0 | 0 | 0 | 0 | 0 |
|  | 1.0 μg/mL | | | | | |
|  | Drug | 2.45 | 2.89 | 2.84 | 2.726667 | 0.22 |
|  | Drug + PDI | 3.5 | 3.52 | 3.67 | 3.563333 | 0.075865 |
|  | PDI + Drug | 0 | 0 | 0 | 0 | 0 |
| Ketoconazole | 0,0625 μg/mL | | | | | |
|  | Drug | 4.3 | 4 | 3.9 | 4.066667 | 0.169967 |
|  | Drug + PDI | 2.1 | 2.2 | 2.5 | 2.266667 | 0.169967 |
|  | PDI + Drug | 1.1 | 1.9 | 2.3 | 1.766667 | 0.498888 |
|  | 0.125 μg/mL | | | | | |
|  | Drug | 4.5 | 4.1 | 4.3 | 4.3 | 0.163299 |
|  | Drug + PDI | 1.9 | 1.9 | 2.3 | 2.033333 | 0.188562 |
|  | PDI + Drug | 0 | 0 | 0 | 0 | 0 |
| Potassium iodide | 7.8 mg/mL | | | | | |
|  | Drug | 4.8 | 4.3 | 4.8 | 4.633333 | 0.235702 |
|  | Drug + PDI | 3.8 | 3.6 | 1.2 | 2.866667 | 1.181336 |
|  | PDI + Drug | 2 | 2 | 2 | 2 | 0 |
|  | 15.6 mg/mL | | | | | |
|  | Drug | 4.5 | 4.1 | 4.5 | 4.366667 | 0.188562 |
|  | Drug + PDI | 3.9 | 3.2 | 3.8 | 3.633333 | 0.309121 |
|  | PDI + Drug | 0 | 1.6 | 2 | 1.2 | 0.864099 |
|  | 31,2 mg/mL | | | | | |
|  | Drug | 4.2 | 3.8 | 4.2 | 4.066667 | 0.188562 |
|  | Drug + PDI | 3.7 | 3 | 3.2 | 3.3 | 0.294392 |
|  | PDI + Drug | 1.4 | 1.4 | 1.6 | 1.466667 | 0.094281 |
|  | 62.5 mg/mL | | | | | |
|  | Drug | 3.9 | 3.7 | 4.2 | 3.933333 | 0.20548 |
|  | Drug + PDI | 3.4 | 2.6 | 3.7 | 3.233333 | 0.46428 |
|  | PDI + Drug | 1.2 | 1.2 | 1.7 | 1.366667 | 0.235702 |
|  | 125 mg/mL | | | | | |
|  | Drug | 3.7 | 3.5 | 4 | 3.733333 | 0.20548 |
|  | Drug + PDI | 3.4 | 2.5 | 3.2 | 3.033333 | 0.385861 |
|  | PDI + Drug | 0 | 0 | 0 | 0 | 0 |

| *S. schenckii* | | | | | | |
| --- | --- | --- | --- | --- | --- | --- |
| Itraconazole | CFU/mL | | | | Average | SD |
|  | 0.0625 μg/mL | | | | | |
|  | Drug | 4.09 | 4 | 4.1 | 4.063333 | 0.045 |
|  | Drug + PDI | 4.17 | 3.81 | 3.9 | 3.96 | 0.18 |
|  | PDI + Drug | 0 | 0 | 0 | 0 | 0 |
|  | 0.125 μg/mL | | | | | |
|  | Drug | 3.96 | 3.37 | 3.5 | 3.61 | 0.253114 |
|  | Drug + PDI | 2.78 | 2.61 | 2.68 | 2.69 | 0.069761 |
|  | PDI + Drug | 0 | 0 | 0 | 0 | 0 |
| Ketoconazole | 0.0625 μg/mL | | | | | |
|  | Drug | 4 | 4 | 4.4 | 4.133333 | 0.188562 |
|  | Drug + PDI | 2.2 | 2 | 3 | 2.4 | 0.432049 |
|  | PDI + Drug | 0 | 0 | 0 | 0 | 0 |
| Potassium iodide | 7.8 mg/mL | | | | | |
|  | Drug | 4.1 | 4.2 | 4.6 | 4.3 | 0.216025 |
|  | Drug + PDI | 4.1 | 3.5 | 2.4 | 3.333333 | 0.703957 |
|  | PDI + Drug | 3.7 | 2.8 | 3.7 | 3.4 | 0.424264 |
|  | 15.6 mg/mL | | | | | |
|  | Drug | 4.1 | 4.1 | 4.3 | 4.166667 | 0.094281 |
|  | Drug + PDI | 2.4 | 2.8 | 4.1 | 3.1 | 0.725718 |
|  | PDI + Drug | 2.33 | 2.5 | 3.7 | 2.843333 | 0.609718 |
|  | 31.2 mg/mL | | | | | |
|  | Drug | 4 | 4 | 4.2 | 4.066667 | 0.094281 |
|  | Drug + PDI | 2.1 | 2.7 | 3.5 | 2.766667 | 0.3 |
|  | PDI + Drug | 0 | 0 | 0 | 0 | 0 |
